# Resilience as the Mediating Factor in the Relationship Between Sleep Disturbance and Post-stroke Depression of Stroke Patients in China: A Structural Equation Modeling Analysis

**DOI:** 10.3389/fpsyt.2021.625002

**Published:** 2021-05-10

**Authors:** Lina Zhao, Fengzhi Yang, Kristin K. Sznajder, Changqing Zou, Yajing Jia, Xiaoshi Yang

**Affiliations:** ^1^Department of English, School of Fundamental Sciences, China Medical University, Shenyang, China; ^2^Department of Social Medicine, School of Public Health, China Medical University, Shenyang, China; ^3^Department of Public Health Sciences, College of Medicine, Pennsylvania State University, Hershey, PA, United States; ^4^Department of Humanities and Social Sciences, China Medical University, Shenyang, China

**Keywords:** sleep disturbance, resilience, post-stroke depression, stroke patients, structural equation modeling

## Abstract

**Background:** Stroke patients may suffer from a variety of symptoms which can result in sleep disturbance and post-stroke depression (PSD). Whereas, resilience can alleviate sleep disturbance and help maintain well-being after stroke.

**Objective:** The aim of this study is to explore whether resilience plays a mediating role in the relationship between sleep disturbance and PSD of stroke patients in China.

**Methods:** A cross-sectional study with a multi-stage sampling was carried out in Liaoning Rehabilitation Center and the Third People's Hospital of Chongqing in China from May to September 2019. A total of 353 stroke patients were enrolled in this study. Structural equation model (SEM) was used to test the mediating effect of resilience on the relationship between sleep disturbance and PSD.

**Results:** The prevalence of PSD of stroke patients was 34.56%. Sleep disturbance contributed most to the variance of PSD and had a significantly positive association with PSD among stroke patients (*P* < 0.01). Resilience was negatively associated with PSD, and acted as a mediator between sleep disturbance and PSD (a ^*^ b = 0.201, BCa 95% CI: 0.156~0.254).

**Conclusions:** The prevalence of PSD was high among the Chinese stroke patients. Sleep disturbance was highly associated with PSD, resulting in the increased risk of PSD. Furthermore, resilience has a mediating effect on the relationship between sleep disturbance and PSD, and could reduce the negative effect of sleep disturbance on the development of PSD.

## Introduction

Stroke is the second cause of death and one of the leading causes of disability in the world (1). It is estimated that there will be 30 million patients suffering from stroke by 2030 (2). Stroke not only damages the physical health of patients, but also affects their mental health. Stroke patients may suffer from a variety of symptoms, including cognitive decline (3), sleep disturbance (4), restricted movement (5) and depression (6). Studies have shown that stroke patients are more likely to suffer from depressive symptoms compared with the general population (7). Post-stroke depression (PSD) has become the most frequent mental disorder among all the symptoms from which stroke survivors suffer. It refers to depression among the patients after stroke resulting from damage of stroke triggered by the symptoms of initial ischemia and reperfusion injury (8). PSD complicates the outcome of stroke recovery, further exacerbates the physical and psychological symptoms, lowers the quality of patients' life and increases the recurrence rate and mortality of stroke (9). Symptoms of PSD include sad mood, slowness in thoughts and emotions, loss of appetite and burnout, and also involve hopelessness, irritability, and sleep problems (10). Patients with PSD have a higher risk of subsequent mortality compared with non-depressed stroke patients. Therefore, PSD is a critical area of study in the field of public health and clinical prognosis.

A large number of studies implied that the prevalence of PSD among stroke survivors was estimated to be from as low as 5% to as high as 63%, while the average prevalence of PSD around the world was ~33% (11, 12). The varied percentage may be due to differences in the definition of stroke, means of PSD assessment, research population, research setting, and stroke stages across different countries (13, 14). The prevalence of PSD in China is estimated to be between 23 and 76% (15). PSD is considered to be a main negative predictor of poor outcomes for physical and social functioning of patients after stroke and is related to physical disabilities, impairment of cognition, and increase of mortality (16). PSD plays a negative role in patients' rehabilitation training, which affects the recovery of function and leads to negative consequences, such as deteriorated cognitive function, increased medical complication rates, and diminished social abilities (17).

Most previous studies found that sleep disturbance was positively related with the prevalence of PSD (18, 19). Sleep disturbance refers to abnormal amount of sleep and abnormal alternation of sleep and wakefulness, and it often occurs after stroke, involves various forms, and interferes with the life of stroke patients' function status (20). The previous studies have implied that the prevalence of depression ranged from 14 to 31% among patients with insomnia (21). Good sleep quality is beneficial to human's daily life, cognitive functions, endocrine function, and thus, sleep disturbance can influence many aspects of the individuals' health, such as physical and emotional health (22). Studies have shown that poor sleep quality is one of the main risk factors of PSD (23, 24), and can negatively affect up to 90% of the depressed patients (25). Stroke patients commonly suffer from sleep disturbances with an estimated prevalence of 40–80% (26, 27), and sleep disturbance is not only associated with stroke patients' rehabilitation outcomes, but also associated with PSD among stroke patients (18). Therefore, sleep disturbance should be considered in the management of those who have suffered from a stroke to relieve PSD.

Based on the Positive Psychology Theory (28), positive psychological resources such as resilience can help relieve symptoms of depression, and help the individuals take adaptive actions to improve their psychological health (29). Resilience refers to the capacity to successfully accommodate to major stressors including tragedy, trauma, adversity and difficulties, which is a dynamic process affected by life events and challenges (30, 31). Low levels of resilience are associated with a greater susceptibility to pathological reactions to negative environmental events for the stroke patients. On the contrary, the patients with high levels of resilience can take advantage of better capability of coping with and adjusting to negative life events adaptively. There are many positive impacts of resilience such as maintaining function, subjective well-being, motivation for activity, and life engagement (32). Previous studies indicated that resilience was an independent predictor of PSD and quality of life in patients with ischemic stroke, and PSD could be mitigated by improved capability of resilience (33, 34). It has also been reported that resilience can help prevent the prevalence of psychiatric disorders in people with chronic diseases (35). However, there is a paucity of research on resilience and PSD in Chinese stroke patients.

There are quite a few studies concerning the associated factors of PSD, however, there are very few studies exploring the effect of positive resource such as resilience on the relationship between sleep disturbance and PSD. Thus, the aims of this study are to elucidate the association between sleep disturbance and PSD of stroke patients, and examine whether the association between sleep disturbance and PSD is mediated by resilience.

## Methods

### Study Design and Participants

A cross-sectional study with multi-stage sampling was implemented in stroke patients in China from May to September 2019. Two steps of multi-stage sampling procedures were employed: at the first-stage, two general hospitals (the Liaoning Provincial Rehabilitation Medical Center and the Third People's Hospital of Chongqing) were randomly selected in the northeast and southwest of China. Cluster sampling was used at the second-stage, all the inpatients with a definite diagnosis of stroke in the cerebrovascular department of each hospital were included in this study. A face-to-face interview was conducted among the stroke patients with rehabilitation treatment in the two hospitals by the trained investigators. The questionnaire pertaining to demographic characteristics, resilience, sleep disturbance, and PSD, which lasted ~15 min, was distributed to the stroke patients. Before the questionnaire was started, informed consent of participants was obtained.

The inclusion criteria of the participants included: (1) ≥18 years old; (2) eligible for the criteria for diagnosis of cerebrovascular disease, with ischemic stroke, cerebral hemorrhage, or subarachnoid hemorrhage confirmed by computer tomography or magnetic resonance imaging, or identified as stroke patients according to clinical manifestations with patients' condition not allowed to be examined or special examinations not available; (3) informed and voluntary to be recruited. Exclusion criteria were: (1) patients whose condition does not meet the above criteria; (2) patients with mental illness or disturbance of consciousness; (3) patients with verbal or intellectual dysfunction with which patients could not complete the questionnaire effectively.

### Ethics Statement

Each participant was well-informed of the aims and contents of the questionnaire. The research was carried out in accordance with the Helsinki Declaration as revised in 1989, and the Ethics Committee of China Medical University approved the protocols of this study (CMU1210400061).

### Measures

#### Demographic Characteristics

Demographic characteristics embodied gender, age, marital status, education, and monthly income. Marital status was divided into three categories, i.e., “single,” “married or cohabitating,” and “divorced, separated, or widow.” Education was categorized as “junior school or lower,” “high school or polytechnic school,” and “undergraduate or higher.” Monthly income was grouped into “ <3,000 RMB,” “3,000~6,000 RMB,” and “>6,000 RMB.”

#### Measurement of PSD

The Patient Health Questionnaire Depression Scale (PHQ-9) with a total of 9 items was designed and validated to diagnose and grade depression on account of DSM-IV criteria (36). Symptoms were assessed on a four-point Likert scale (0 = Never; 1 = Several days; 2 = More than half of the time; and 3 = Nearly every day) with a range in total score from 0 to 27 based on the experience for the past 2 weeks. A cutoff of 10 or greater has been described as diagnostic in systematic reviews and meta-analysis of the PHQ-9 and was used as the cutoff for the diagnosis of PSD in this study (37). The PHQ-9 has a good reliability, with the Cronbach's alpha coefficient of 0.90 for this scale.

#### Measurement of Sleep Disturbance

The PROMIS Sleep Disturbance Short Form was used to evaluate the severity of stroke patients' sleep disturbance in the past seven days (38). This scale consisted of 8 items, which were scored on 5-point Likert scale (1 = Never; 2 = Rarely; 3 = Sometimes; 4 = Often; and 5 = Always). Responses to each item were summed to get the total original scores, which ranged from 8 to 40. Then, the original score was converted to a standardized T-score, with higher scores indicating greater severity of sleep disturbance (39). The Cronbach's alpha coefficient of this scale was 0.96 in this study, which indicated that it had a good reliability.

#### Measurement of Resilience

The Ego-Resiliency Scale was employed to measure the trait of psychological resilience (40). It included 14 items such as “I quickly get over and recover from being startled” and was developed to assess flexibility, curiosity, generosity and social skills. A 4-point Likert scale (1 = Does not apply at all; 2 = Applies slightly; 3 = Applies somewhat; and 4 = Applies very strongly) was adopted to measure each item. The total score ranged from 14 to 56, with higher scores indicating greater ego-resiliency. This scale has been widely used in previous studies in China with a good reliability (41). The Cronbach's alpha coefficient of this scale was 0.93 in this study.

### Statistical Analysis

Two independent sample *t*-test and one way analysis of variance (ANOVA) were used to compare the difference of PSD among categorical variables. In this study, two independent sample *t*-test was used to compare the difference of PSD among two categorical variables, including age, gender, and marital status. One way ANOVA was used to explore the difference of PSD among the multiple categorical variables, including education and monthly income. Pearson correlation was conducted to examine the correlations of sleep disturbance, resilience, and PSD. Hierarchical Multiple Regression (HMR) analysis was performed to explore the predictors of PSD and test the mediating effect of resilience on the relationship between resilience and PSD. The asymptotic and resampling strategies developed by Preacher and Hayes were used in this study to examine resilience as a potential mediator on the association between sleep disturbance and PSD (42). PSD was used as a dependent variable. The independent variables were entered in three steps as follows: Step 1: demographic characteristics of stroke patients; Step 2: sleep disturbance; and Step 3: resilience. If the regression coefficient of sleep disturbance to the PSD was significant and decreased from step 2 to step 3, there was a partial mediated effect. If the regression coefficient was not statistically significant (*P* > 0.05), a complete mediating role of resilience was indicated. The analysis was performed in stages by successively inputting blocks of independent variables in the model. In addition, Structural Equation Model (SEM) was used to confirm the mediating effect of resilience on the relationship between sleep disturbance and PSD (43) which was analyzed by Amos 17.0. The model fitted with the SEM criteria (χ^2^/df < 5, GFI > 0.90, CFI > 0.90, RMSEA < 0.08, and TLI > 0.90). Sober test was employed to test whether the mediating effect was statistically significant (44). Bootstrapping was employed to examine the mediator (a^*^b product) of resilience on the relationship between sleep disturbance and PSD, with the estimate of 5,000 samples. A bias-corrected and accelerated 95% CI (BCa 95% CI) for each a^*^b product was examined. All analyses were performed using SPSS version 21.0 and Amos 17.0 statistical software for Windows. Statistical significance was defined as *P* < 0.05 (two-tailed).

## Results

### Demographic Characteristics

The stroke patients' demographic characteristics are presented in Table 1. The average age of stroke patients was 57 ± 14 years old. Among the 353 patients, 240 (67.99%) were males and 113 (32.01%) were females. The majority (92.92%) of the stroke patients were married or cohabiting. About 42.78% of the stroke patients had an educational level of junior school or below, and nearly one-fifth (19.26%) of the stroke patients had a monthly income level of more than 6,000 RMB, while 45.33% of them had a monthly income of 3,000–6,000 RMB. PSD scores were significantly higher in stroke patients with the ages of over 60 than in those who were 60 or under, and PSD scores were negatively associated with monthly income. Sleep disturbance scores were significantly higher in stroke patients with the ages of over 60 and monthly income <3,000 RMB. Age, education, and monthly income were all associated with resilience scores, and stroke patients aged ≤60 had higher resilience scores compared to those with the ages more than 60. Also, stroke patients with undergraduate or higher education level reported higher resilience scores than other groups. Stroke patients whose monthly income >6,000 RMB had higher resilience than those with a monthly income of 3,000–6,000 RMB and <3,000 RMB. The mean score of PSD among the stroke patients in the present study was 8.84 and the prevalence of PSD was 34.56%.

**Table 1:** Demographic characteristics and distributions of PSD, sleep disturbance, and resilience among stroke patients.

**Variables**	***N* (%)**	**PSD(Mean ± SD)**	**Sleep disturbance (Mean ± SD)**	**Resilience(Mean ± SD)**
**Age (years)**				
≤ 60	185 (52.41)	7.66 ± 4.92	46.68 ± 8.99	33.44 ± 8.50^**^
>60	168 (47.59)	10.13 ± 5.87^**^	52.41 ± 10.35^**^	29.96 ± 8.77
**Gender**				
Male	240 (67.99)	8.83 ± 5.31	49.37 ± 10.14	32.25 ± 8.78
Female	113 (32.01)	8.86 ± 5.97	49.47 ± 9.95	30.81 ± 8.77
**Marital status**				
Married or cohabiting	328 (92.92)	8.97 ± 5.44	49.67 ± 10.11	31.57 ± 8.65
Others	25 (7.08)	7.12 ± 6.34	45.90 ± 8.89	34.60 ± 10.23
**Education**				
Junior school or below	151 (42.78)	8.96 ± 5.64	50.63 ± 10.38	30.32 ± 8.62
High school or polytechnic school	123 (34.84)	8.87 ± 5.36	48.09 ± 9.78	31.85 ± 8.21
Undergraduate or higher	79 (22.38)	8.56 ± 5.61	49.12 ± 9.71	34.48 ± 9.42^**^
**Monthly income (RMB)**				
<3,000	125 (35.41)	10.24 ± 5.79^**^	52.25 ± 11.30^**^	27.89 ± 8.51
3,000–6,000	160 (45.33)	8.16 ± 5.12	47.82 ± 8.87	33.26 ± 8.15
>6,000	68 (19.26)	7.85 ± 5.50	47.91 ± 9.23	35.50 ± 8.18^**^

** *P < 0.01*.

### Correlations among Sleep Disturbance, Resilience, and PSD

**Figure 1:**
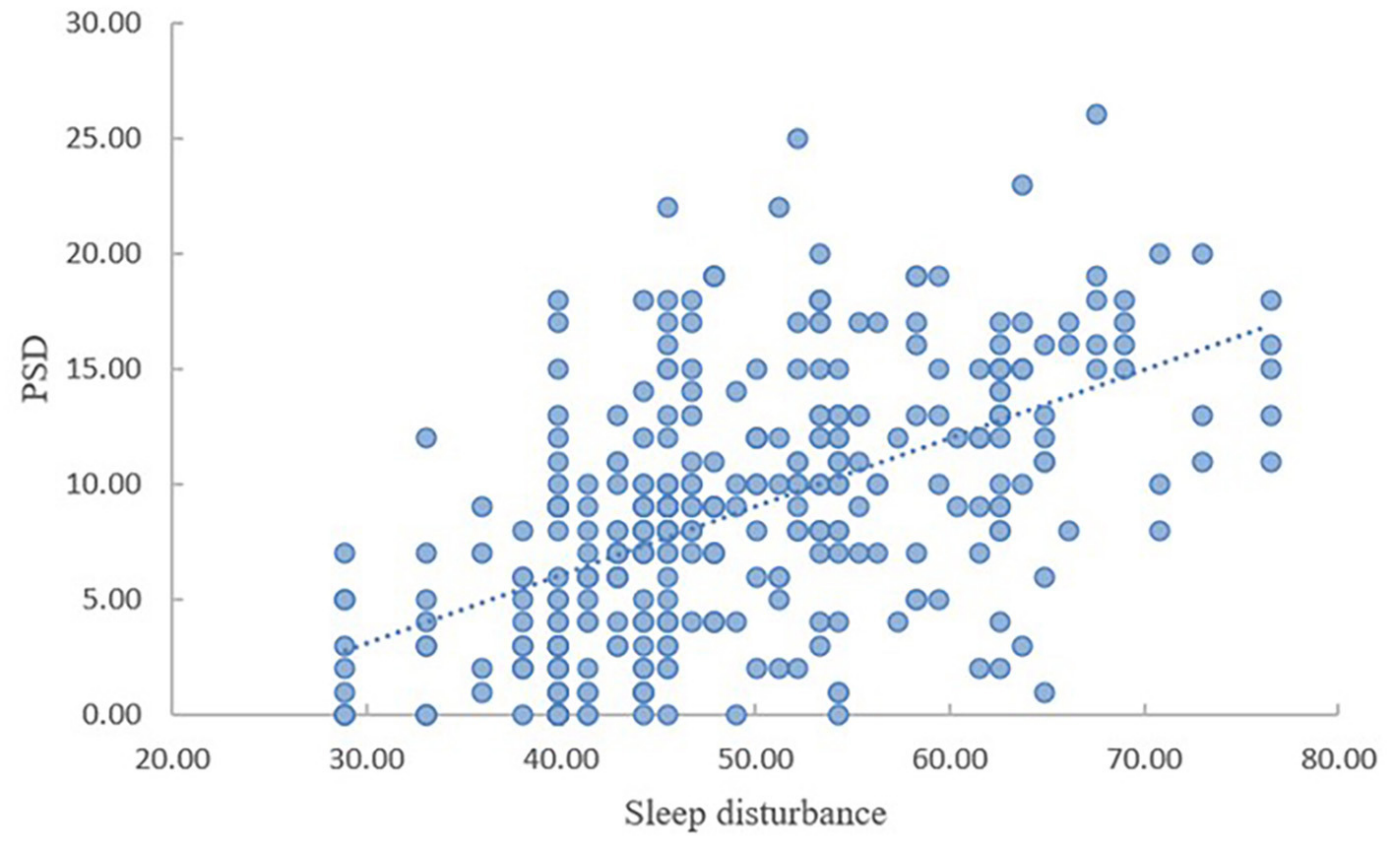
The correlation between sleep disturbance and PSD.

**Figure 2:**
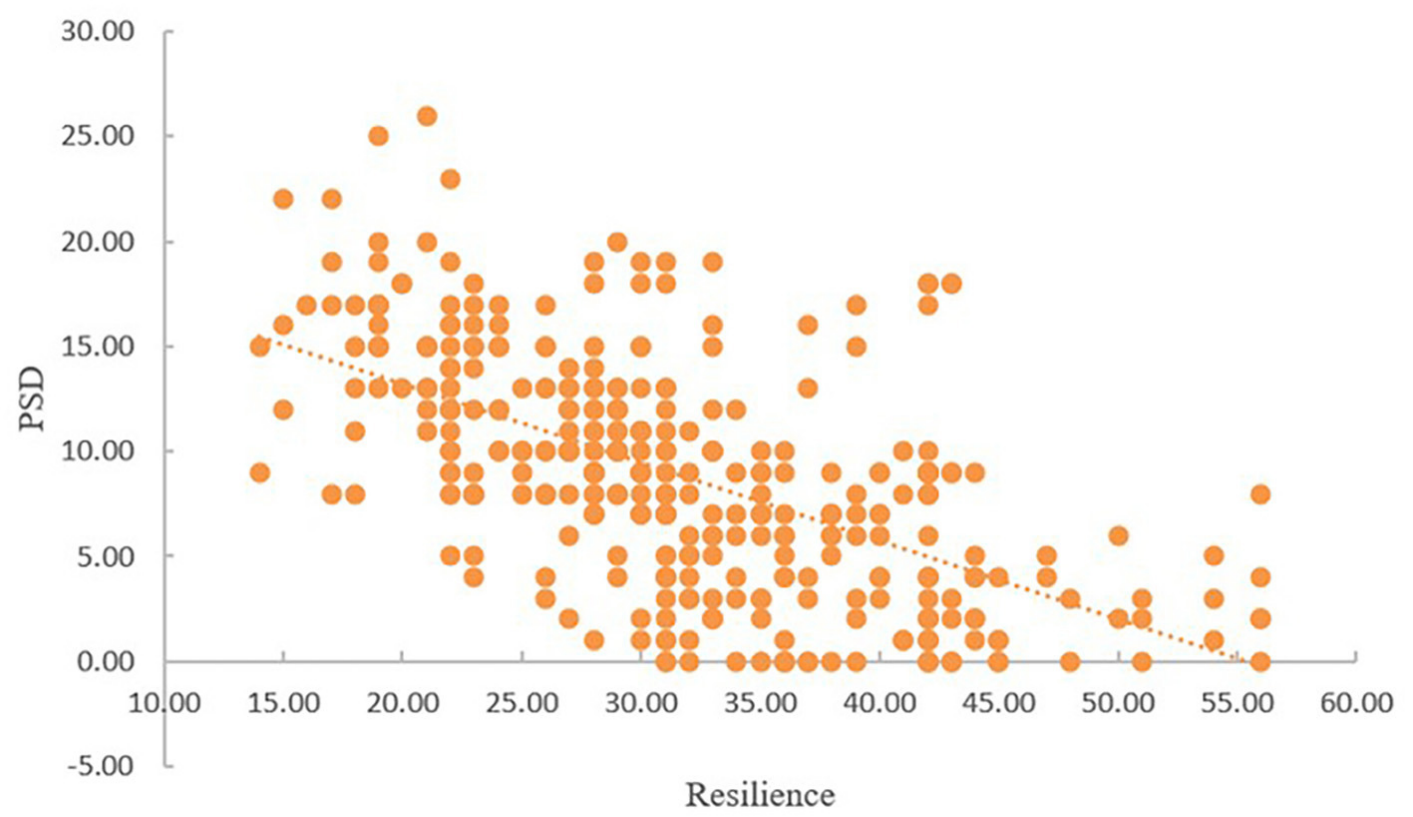
The correlation between resilience and PSD.

As shown in Table 2, PSD was significantly linked with sleep disturbance and resilience. Sleep disturbance was negatively correlated with resilience (*r* = −0.398, *P* < 0.01), while it was positively correlated with PSD (*r* = 0.528, *P* < 0.01). There was a negative correlation between resilience and PSD (*r* = −0.594, *P* < 0.01). As presented in Figures 1, 2, with the increase of sleep disturbance score, the score of PSD also increased, while with the increase of resilience score of stroke patients, the degree of PSD gradually decreased, which indicated that sleep disturbance was positively correlated with PSD in stroke patients, and resilience was negatively correlated with PSD, which was consistent with the results of Table 2.

**Table 2:** The Pearson correlation among age, sleep disturbance, resilience, and PSD.

**Variable**	**1**	**2**	**3**	**4**
PSD	1			
Age	0.293^**^	1		
Sleep disturbance	0.528^**^	0.319^**^	1	
Resilience	−0.594^**^	−0.257^**^	−0.398^**^	1

** *P < 0.01*.

### Regression Analysis of Sleep Disturbance, Resilience, and PSD

The HMR models of PSD are depicted in Table 3. The final HMR model explained a total of 46.8% of the variance in PSD. Age and monthly income were significantly associated with PSD in model 1. According to the *R*^2^ change, sleep disturbance contributed most to the total variance of PSD (21.4%), and resilience was responsible for 15.7% of total variance of PSD. Sleep disturbance and resilience were the strong predictors of PSD. Additionally, PSD was positively associated with sleep disturbance and negatively associated with resilience.

**Table 3:** The hierarchical multiple regression models of PSD.

		**Model 1**			**Model 2**			**Model 3**	
	***B***	**β**	**95%CI**	***B***	**β**	**95%CI**	***B***	**β**	**95%CI**
Block 1 Demographic characteristics									
Age (≤ 60 years vs. >60 years)	0.221^**^	0.221^**^	0.119~0.323	0.084	0.084	−0.009~0.177	0.046	0.046	−0.036~0.128
Gender (Male vs. Female)	−0.020	−0.020	−0.122~0.083	−0.005	−0.005	−0.095~0.085	−0.022	−0.022	−0.101~0.057
Marital status (Married or cohabiting vs. others)	0.084	0.084	−0.017~0.186	0.038	0.038	−0.051~0.127	0.009	0.009	−0.069~0.088
**Education**									
(Junior school or below vs. High school or polytechnic school)	0.077	0.077	−0.039~0.193	0.096	0.096	−0.005~0.197	0.072	0.072	−0.017~0.161
(Junior school or below vs. Undergraduate or higher)	0.089	0.089	−0.033~0.211	0.063	0.063	−0.044~0.170	0.082	0.082	−0.013~0.176
**Monthly income**									
(<3,000 RMB vs. 3,000–6,000 RMB)	−0.212^**^	−0.212^**^	−0.330~-0.094	−0.103	−0.103	−0.208~0.002	0.001	0.005	−0.094~0.096
(<3,000 RMB vs. >6,000 RMB)	−0.212^**^	−0.212^**^	−0.336~0.088	−0.121^*^	−0.121^*^	−0.231~-0.012	0.005	0.001	−0.095~0.105
Block 2 Sleep disturbance				0.498^**^	0.498^**^	0.404~0.593	0.345^**^	0.345^**^	0.257~0.434
Block 3 Resilience							−0.459^**^	−0.459^**^	−0.549~-0.369
*R*^2^	0.097			0.311			0.468		
Adjust*R*^2^	0.078			0.295			0.454		
Δ*R*^2^	0.097			0.214			0.157		

* *P < 0.05*,

** *P < 0.01*.

### Mediator of Resilience Between Sleep Disturbance and PSD

**Figure 3:**
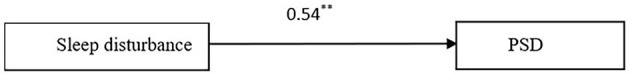
Standardized solutions for the structural equation model of sleep disturbance and PSD. **Indicating the coefficient of the path is significant.

The direct pathway between sleep disturbance and PSD is illustrated in Figure 3. The SEM model showed sleep disturbance had a significant direct influence on PSD (*c* = 0.54, *P* < 0.01). The SEM implied that sleep disturbance was positively associated with PSD and this model had good model fit indices (χ^2^/df < 5, RMSEA = 0.069, CFI = 0.972, GFI = 0.956, AGFI = 0.922, and TLI = 0.959). The Sobel test was used to confirm the statistical significance of mediating effects. For the indirect effect, the results of Sobel test indicated the significant mediating effect of resilience on the relationship between sleep disturbance and PSD.

**Figure 4:**
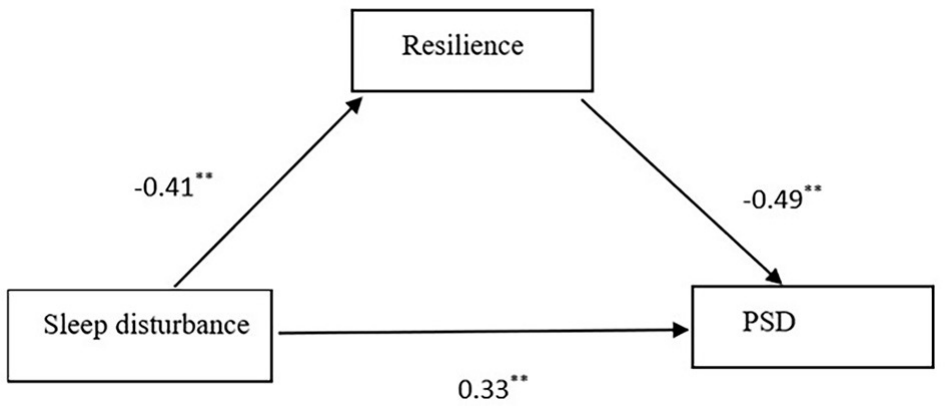
Standardized solutions for the structural equation model of resilience, sleep disturbance, and PSD. **Indicating the coefficient of the path is significant.

The mediating role of resilience in the association between sleep disturbance and PSD is shown in Figure 4. Resilience was significantly and negatively associated with PSD (β = −0.49, *P* < 0.01). Moreover, when resilience was modeled as a mediator, the path coefficient of sleep disturbance on PSD decreased significantly (c′ = 0.33, *P* < 0.01). The bias-corrected and accelerated bootstrap test indicated that resilience significantly mediated the relationship between sleep disturbance and PSD (a ^*^ b = 0.201, BCa 95% CI: 0.156~0.254), which confirmed a significant partial mediating role of resilience in the association between sleep disturbance and PSD. The model presented in Figure 4 was fully supported by all standard goodness of fit indices (χ^2^/df < 5, RMSEA = 0.062, CFI = 0.975, GFI = 0.957, AGFI = 0.925, and TLI = 0.963). Thus, sleep disturbance directly affected PSD and also influenced PSD indirectly by the mediating pathway of resilience.

## Discussion

To the best of our knowledge, the present study represents the initial attempt to examine the mediating role of resilience in the relationship between sleep disturbance and PSD among stroke patients. The prevalence of PSD among stroke patients in this study was 34.56%, which was higher than that among stroke survivors in a systematic review (29%) (45). Chinese stroke patients exerted high levels of PSD, and sleep disturbance could result in the elevated risk of PSD. However, resilience, acting as the mediator in the relationship between sleep disturbance and PSD, could relieve the negative effects of sleep disturbance on PSD. Neither psychological counseling nor treatment was provided to the Chinese stroke patients in the general hospitals recently, and rehabilitation mostly focused on the recovery of stroke patients' physical health, with their mental health being neglected. This study revealed that the older patients and the patients with low incomes had more serious symptoms of PSD, which was in accordance with previous studies (46, 47). This may be because elder patients are usually accompanied with disabilities or chronic diseases, with decreased physiological function, with the need of family care in the long run, or with the guilt to family, which finally increases the risk of PSD. This study showed that resilience and sleep disturbance had significant direct effects on PSD. Furthermore, resilience could partially mediate the relationship between sleep disturbance and PSD.

It was found that sleep disturbance contributed the most to PSD in this study, which was in agreement with most previous studies showing that sleep disturbance was positively associated with depression in patients with heart disease (48), lupus (49), and chronic obstructive pulmonary disease (50). PSD is the most common clinical complication and sequelae of stroke, studies have shown that the levels of IL-6, IL-1β, and Hcy in patients with PSD are significantly higher than those in the normal population (51). There were also studies demonstrating that iNOS, MIP-1α levels and miRNAs were associated with PSD (52, 53). Besides, sleep quality of stroke patients was also an important influencing factor of PSD (54). Sleep disturbance is the most common complication during the stroke recovery period and most stroke patients who suffer from sleep disturbance are likely to have an increased risk of depression (55, 56). This study revealed that the more serious the sleep disturbance was, the more severely the stroke patients suffered from PSD. There is escalating evidence showing that sleep disturbance is associated with stroke occurrence and prognosis, while adequate sleep may contribute to neuroprotection and promotion of recovery of post-stroke functioning of nervous system (57). Stroke patients, frequently confronted with difficulties in falling asleep and maintaining sleep (58), which are associated with PSD, will have compromised recovery ability after stroke and be always with a poorer prognosis. Poor sleep quality could affect patients' cognition and emotion processes, resulting in changes of behaviors featured by more irritability and unstable emotion (59), which could engender poor mental health, presented by the symptoms of PSD.

A significantly negative association between resilience and PSD was found in this study, which indicated that a high level of resilience could reduce the prevalence of PSD. Resilience is a personality-like and state-like trait that can enable individuals to maintain mental health and improve coping capacities when exposed to stressful situations (60), and individuals with low levels of resilience may be more susceptible to pathological reactions to negative life events (61). It was found in this study that the resilience of stroke patients was lower than that of the general population. Previous studies demonstrated that resilience could prevent and treat various psychiatric disorders (34, 62), and stroke patients with more resilience might recover more quickly from sad mood and sleep disturbance, which could enhance stroke patients' abilities to adapt to the process of rehabilitation training in hospitals and maintain optimal well-being. Thus, resilience enhancement should be provided for the stroke patients in order to alleviate the symptoms of PSD.

Notably, sleep disturbance not only had a direct effect on PSD, but also had an indirect effect on PSD through the mediating path of resilience in this study. The relationship between sleep disturbance and PSD among stroke patients confirmed that greater sleep disturbance was associated with a higher risk of PSD. This study found that PSD might deteriorate among stroke patients who had sleep disturbance, but this association might be reversed by improved resilience. Resilience could effectively regulate sleep quality, attenuate the detrimental impacts of sleep disturbance on mental health, and thus relieve symptoms of PSD. Psychological resources like resilience can directly affect depressive episodes, as well as indirectly affect depression through increased social support and effective coping methods (29). Some studies have shown that resilience plays a mediating role in the relation between social support and depression (63). Resilience could be protective against PSD, facilitate patients' ability to rationally combat the harmful effect of sleep disturbance and enhance their abilities to promote mental health status. Therefore, resilience training should be emphasized for stroke patients in order to combat the stressful situations to promote the recovery of rehabilitation and enhance the mental health status and well-being for the stroke patients.

## Conclusion

The prevalence of PSD was significantly high among the Chinese stroke patients (34.56%) in this study. Sleep disturbance was highly associated with PSD, whereas, resilience was negatively associated with PSD. Furthermore, resilience played a mediating role in the relationship between sleep disturbance and PSD, and could reduce the negative effect of sleep disturbance on the development of PSD. Sleep quality promoting and resilience training for stroke patients are necessary and should be valued and strengthened to reduce the prevalence of PSD and to improve their rehabilitation of physical function and maintain their mental well-being.

## Limitation

A few limitations exist in the present study. Firstly, the cross-sectional design in this study cannot establish causal relationships among study variables. Secondly, only two hospitals were selected in this study, which might limit the generalizability of the study. Finally, the results of this study might be limited by unmeasured confounders such as individual's social status and regional difference in health level.

## Data Availability Statement

The original contributions presented in the study are included in the article/supplementary material, further inquiries can be directed to the corresponding author.

## Ethics Statement

The studies involving human participants were reviewed and approved by the Ethics Committee of China Medical University (CMU1210400061). The patients/participants provided their written informed consent to participate in this study.

## Author Contributions

LZ contributed to acquisition and analysis of data, drafting, and revision of the manuscript. FY contributed to acquisition and interpretation of data and revision of the manuscript. KS contributed to revision of the manuscript and provided English edits. CZ contributed to acquisition of data and revision of the manuscript. YJ contributed to acquisition and interpretation of data. XY was responsible for the conception and design. All authors read and approved the final manuscript.

## Conflict of Interest

The authors declare that the research was conducted in the absence of any commercial or financial relationships that could be construed as a potential conflict of interest.
